# The effect of uninterrupted and interrupted sitting on vascular function in adults with long COVID

**DOI:** 10.14814/phy2.70452

**Published:** 2025-10-06

**Authors:** Nick Hudson, Scott Hannah, Margaret Husted, Simon Fryer, Helen Ryan‐Stewart, Mark Rickenbach, Keeron Stone, James Faulkner

**Affiliations:** ^1^ School of Sport, Health and Community University of Winchester Winchester UK; ^2^ School of Psychology University of Winchester Winchester UK; ^3^ School of Education and Science University of Gloucestershire Gloucester UK; ^4^ Faculty of Education, Humanities and Health Sciences EIT | Te Pūkenga Hawke's Bay New Zealand; ^5^ Faculty of Health and Wellbeing University of Winchester Winchester UK; ^6^ Park and St Francis Surgery Eastleigh, Hampshire UK; ^7^ Centre for Cardiovascular Research, Innovation and Development (CURIAD), Cardiff School of Sport and Health Sciences Cardiff Metropolitan University Cardiff UK; ^8^ National Cardiovascular Research Network Cardiff UK; ^9^ Primary Care Research Centre University of Southampton Southampton UK

**Keywords:** arterial stiffness, blood pressure, COVID‐19, Long COVID, pulse wave velocity

## Abstract

Acute prolonged sitting increases blood pressure (BP) and arterial stiffness (AS). Both of these may be mitigated via light physical activity (LPA). Whether long COVID (LC), which partly manifests as vascular sequelae, predisposes a heightened sensitivity to sitting or diminished benefits from its interruption is unknown. The aims of this study were to identify whether individuals with LC: (i) exhibit a worse BP/AS response to uninterrupted sitting and (ii) a diminished mitigation of BP/AS response to sitting interrupted with LPA, compared to healthy controls. Thirty participants with LC and 15 controls completed 2 h of uninterrupted sitting and sitting interrupted with LPA. Central and peripheral systolic and diastolic BP and carotid‐femoral pulse wave velocity (cfPWV) were determined pre and post sitting. Linear mixed‐effects models demonstrated no three‐way or two‐way interactions for any variable. There was a significant main effect of time, with increases in central systolic (MD = 3.37 mmHg, SE = 0.93 mmHg, *p* < 0.001) and central diastolic (MD = 3.00 mmHg, SE = 0.58 mmHg, *p* < 0.001) BP. cfPWV was not altered in sitting in either group (MD = 0.13 m/s, SE = 0.09 m/s, *p* = 0.170). Uninterrupted sitting increases BP similarly, but AS is unchanged. Interrupting sitting with LPA did not mitigate sitting‐induced increase in BP regardless of LC diagnosis.

## 1 INTRODUCTION

Long COVID (LC) is a chronic condition driven by persistent inflammation (Raveendran et al., 2021) following acute infection of the SARS‐CoV‐2 virus, more commonly referred to as COVID‐19. The symptoms of LC, notably fatigue (Aiyegbusi et al., 2021), disrupt activities of daily living, including increasing time spent in sedentary behaviors (SB) (Humphreys et al., 2021; Wright et al., 2022) defined as any waking behavior in a seated, reclined, or lying posture and low energy expenditure (Tremblay et al., 2017). Engaging in SB contributes to a heightened risk of cardiovascular disease and all‐cause mortality (Ekelund et al., 2019).

The relationship between SB and cardiovascular risk is partly mediated by the effects of prolonged sitting on blood pressure (BP) and arterial stiffness. Meta‐analyses of studies involving healthy individuals reported that uninterrupted prolonged sitting of 1 h can acutely increase blood pressure (BP) and central arterial stiffness (Adams et al., 2023; Paterson et al., 2022). Both elevated BP and AS are strong predictors of future CVD risk (Fuchs & Whelton, 2020; Vlachopoulos et al., 2010). Elevated BP is likely driven by a combination of blood pooling resulting in a reduction in venous return, and an increase in peripheral resistance (Charkoudian & Rabbitts, 2009; Gordan et al., 2015; Hall et al., 2011; Shvartz et al., 1983; Tansey et al., 2019). Arterial function can become impaired (Paterson et al., 2020) as sitting induces a reduction in blood flow and shear stress, subsequently attenuating endothelium‐derived vasodilator nitric oxide availability (Ogoh, 2023; Stoner et al., 2020). A reduction in nitric oxide leads to a decrease in arterial elasticity, the reciprocal of arterial stiffness. These disruptions are likely compounded by several detrimental autonomic, hormonal, and metabolic factors (Stoner et al., 2021). While prolonged sitting induces vascular changes in healthy populations (Paterson et al., 2020, 2022), interrupting prolonged sitting periods with light physical activity (LPA) such as walking and simple resistance activities can attenuate impairments in vascular function (Paterson et al., 2020, 2022).

Angiotensin converting enzyme 2 (ACE‐2), which plays an important role in cardiovascular function (Beyerstedt et al., 2021), serves as a functional receptor of SARS‐CoV‐2 and is highly expressed in the epithelium of blood vessels (Guney & Akar, 2021). Downregulation of ACE‐2 and inflammatory responses lead to poor vascular health (Banu et al., 2020; Santos et al., 2005), following acute infection (Jannasz et al., 2023; Oikonomou et al., 2022), and in those with LC up to at least 13 months following acute infection (Nandadeva et al., 2023). People with pre‐existing poor vascular health, such as those with hypertension, are known to exhibit a heightened vascular response to prolonged sitting (Dempsey et al., 2018). Consequently, it is plausible that individuals with LC may be predisposed to an increased sensitivity to sitting or experience diminished benefits from interrupting prolonged sitting.

The purpose of the present study was to identify whether individuals with LC exhibit: (i) a worse BP or AS response to uninterrupted sitting, and (ii) a diminished mitigation of BP or AS response to sitting interrupted with LPA, compared to healthy controls.

## 2 METHODS

This study is reported in accordance with the CONSORT (Consolidated Standards of Reporting Trials) guidelines (CONSORT, 2015). The study received approval from the Health Research Authority (HRA) and Health and Care Research Wales (HCRW), reference 22/SC/0120.

### 2.1 Study design

This study utilized a mixed‐factorial design with two independent groups: individuals with long COVID (LC) and healthy (non‐LC) controls (HC). Each group underwent two experimental conditions—interrupted sitting and uninterrupted sitting—in a randomized order using an online random number generator, where 1 = uninterrupted visit first, 2 = interrupted visit first (https://g.co/kgs/cKfrnjw). Experimental order was concealed to participants until the beginning of their first experimental visit. Repeated measurements were taken at two time points: pre‐ and post‐condition to assess vascular health and function. Participants attended the laboratory on three separate occasions. The first visit was used for gaining written consent, taking anthropometric measurements, and familiarizing participants with the experimental procedures. LC participants were given validated questionnaires to assess health‐ and symptom‐related outcomes, and all participants wore an activity monitor for 7 days prior to experimental visits. The following two experimental visits were scheduled 7 days after the familiarization session, separated by at least 72 h (typically 7 days), with a maximum of 30 days between visits. For the experimental visits, participants attended the laboratory following a 3‐h fast (with water permitted), having abstained from caffeine for 12 h and from engaging in strenuous PA for 24 h.

### 2.2 Participants

Recruitment occurred through four GP clinics, two LC clinics, and social media advertisements. Medical diagnosis of LC was confirmed via GP letter, identification through GP database, or confirmation of attendance to an NHS long COVID clinic. Eligibility criteria included participants who were nonsmokers, free from CVD, diabetes, and without recent positive COVID‐19 infection (within the previous 6 weeks for healthy controls, 4 weeks for the LC group) identified by polymerase chain reaction (PCR) or lateral flow test. A health history questionnaire was administered to ensure eligibility. Data were collected at the University of Winchester and University of Gloucestershire (UK) within a thermo‐neutral exercise physiology laboratory.

### 2.3 Patient and public involvement and engagement (PPIE)

LC patients and public representatives (*n* = 2) were actively involved in the development of the study design and the preparation of participant information materials (i.e., consent form and participant information sheet). Their feedback ensured the feasibility and appropriateness of the interruption task and ensured that study documentation was understandable to a nonspecialist audience.

### 2.4 Familiarization

Informed consent was obtained, and participants were familiarized with experimental procedures. Body mass index (BMI) was calculated through assessment of height (Seca, 213, Germany) and mass (Seca, Quadra 808, Germany). LC participants completed the COVID‐19 symptom score and EQ‐5D‐5L (Janssen et al., 2013). These questionnaires identify the type and severity of symptoms displayed by participants and their baseline well‐being.

### 2.5 Intervention (uninterrupted sitting; interrupted sitting)

#### 2.5.1 Uninterrupted sitting

**FIGURE 1:**
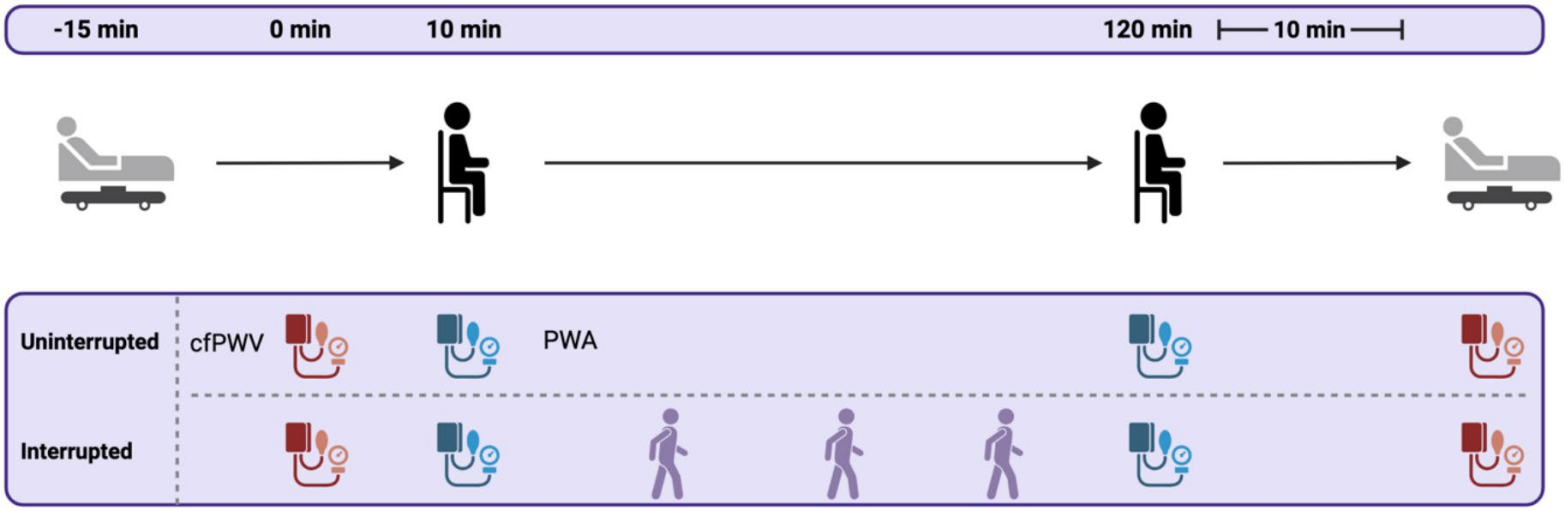
Schematic of procedures—created with BioRender.com

Participants completed the EQ‐5D‐5L prior to procedures. Participants were required to lay supine for 15 min in accordance with recommended guidelines (Paterson et al., 2023) (Figure 1—Schematic of procedures). During this time, brachial and femoral oscillometric cuffs were placed on the upper left arm and thigh of the participant, and cfPWV was measured. Participants then transitioned to a seated position one step away from the plinth where PWA was taken, and they began their 120‐min sitting period. They remained seated with feet touching the floor and a 90‐degree knee angle to minimize muscle contraction for 120 min. During the sitting period, participants could watch a low‐stimulus documentary or read a book and were provided with a low‐fat protein bar (Lean Protein Bar, MyProtein) at the beginning of the session. Participants were instructed to refrain from fidgeting or lower‐limb movement. PWA was initially measured 10 min into the 120‐min sitting period. At 120 min, PWA was repeated in the seated position. Participants then returned to the supine position, and cfPWV was repeated after a further 10 min of rest. A researcher remained present throughout to ensure compliance. A telephone call 72 h post‐visit allowed researchers to check participant welfare following their visits. Should symptoms have been exacerbated due to the study, weekly follow‐up phone calls would have taken place up to 30 days, where participants would have been withdrawn (if following experimental visit 1) and their GP contacted. No participant GP was required to be contacted.

#### 2.5.2 Interrupted sitting

The interruption protocol was similar to the aforementioned uninterrupted sitting protocol and the EQ‐5D‐5L was readministered; however, participants took part in 3 × 5‐min bouts of light activity, approximately every 30 min during the 120‐min sitting period (Figure 1). Each bout of activity included five sit‐to‐stands, five bilateral calf raises, and 3 min of self‐paced walking. With the latter, participants were asked to walk within the laboratory on a flat surface between two cones 10 m apart for up to 3 min.

### 2.6 Outcomes

#### 2.6.1 Pulse wave velocity

Pulse wave velocity (PWV) is the speed at which the forward pressure wave propagates along the arterial tree. The gold standard for assessing PWV is carotid‐femoral PWV (cfPWV), which reflects central (aortic) arterial stiffness (Laurent, 2008; Mitchell et al., 2010; Nagai et al., 1999; Van Bortel et al., 2012). The Sphygmocor XCEL device (AtCor Medical, Sydney, Australia) was used to measure cfPWV according to manufacturer and recommended guidelines (Butlin et al., 2013). A tonometer was placed on the left carotid artery, and an oscillometric cuff was placed over the left femoral artery. PWV is calculated by dividing arterial path length by pulse transit time. Arterial path length is measured using the distance from the sternal notch to the top edge of the femoral (thigh) cuff (distal distance) and from the carotid artery to the suprasternal notch (proximal distance). The proximal distance is subtracted from the distal distance to provide the aortic distance. The additional segment of the femoral pulse to the femoral cuff and the calculated transit time for the pulse to travel this distance are subtracted from the PWV equation to allow for an accurate vascular path length. cfPWV was conducted by triplicate as a minimum and quadruplicate if variability was >0.5 m/s (Townsend et al., 2015). The average of the closest two values was used for analysis.

### 2.7 Blood pressure and pulse wave analysis (PWA)

The SphygmoCor Xcel was used to conduct PWA assessments pre and post 120 min of sitting. The upper arm cuff was initially inflated for approximately 30 s to measure brachial systolic blood pressure (SBP) and diastolic blood pressure (DBP), and then immediately reinflated to 10 mmHg below DBP to acquire a volumetric displacement signal for 10 s. The brachial waveforms were calibrated using the cuff measured SBP and DBP, and mean arterial pressure (MAP) was derived by integrating the area under the curve. A corresponding aortic pressure waveform was generated using a validated transfer function and calibrated using DBP and MAP (Butlin et al., 2012). The aortic waveform was used to derive central: systolic blood pressure (cSBP), diastolic blood pressure (cDBP), mean arterial pressure (MAP), pulse pressure (cPP), augmentation pressure (cAP), augmentation index (AIx), augmentation index normalized to a heart rate of 75 bpm (AIx@75), forward aortic pressure (Pf), backward aortic pressure (Pb) and reflection magnitude (RM) were derived. The Artery Task Force suggests central blood pressure may be influenced by respiration by 2–4 mmHg (Sharman et al., 2017). As such, all PWA assessments were conducted in triplicate as a minimum, and quadruplicate if variability in cSBP was >5 mmHg. For all PWA variables, the average of the closest two was used for all analyses. Each PWA assessment was separated by a 1‐min period.

### 2.8 Habitual physical activity

The ActivPal (activPAL4, Pal Technologies Ltd., Glasgow, United Kingdom) is a valid and reliable tool for measuring SB (Kozey‐Keadle et al., 2011). During familiarization, the ActivPal was placed one‐third of the way between the hip and knee of the right thigh and covered in a finger sleeve and water‐resistant adhesive (Tegaderm, 3M). This remained on for 7 days. Participants were instructed to perform their usual weekly activity and could shower, bathe, and swim with the monitor. Data were deemed valid and included in the analysis if a minimum of 5 valid days (including one weekend day) was reached (Aguilar‐Farias et al., 2018). Data were analyzed using PALanalysis software (Pal Technologies LTD, Glasgow, United Kingdom) to determine step count, activity level (Metabolic equivalents per hour per day (MET·h^−1^/day), calculated by average MET level per hour × wear time in hours), sedentary time, and seated time before being subsequently analyzed in Jamovi (Version 2.5). Twenty‐nine participants' data were deemed to be valid. Non‐validity was due to equipment error/malfunction.

### 2.9 EQ‐5D‐5L

The EQ‐5D‐5L is a validated questionnaire to assess health (Janssen et al., 2013). It evaluates health over five different dimensions: mobility, self‐care, usual activities, pain/discomfort, and anxiety/depression. Each dimension is assessed through a Likert scale detailing five possible answers. The responses can be summarized as a five‐number string, which is an EQ‐5D profile. There are 3125 possible different profiles, for example, No problems on any dimension = 11,111, unable to/extreme problems on all dimensions = 55,555, moderate problems on all dimensions = 33,333. The questionnaire includes a visual analogue scale which captures respondents' overall assessment of their health on a scale from 0 (worst possible health you can imagine) to 100 (best possible health you can imagine).

### 2.10 Sample size

Using the effect size of *d* = 0.36, derived from the main effect of change in cfPWV between pre‐ and post‐sitting from previous literature (Credeur et al., 2019) and the maximum chances of type 1 error set at 5% and power set at 0.95, the number of participants required was 24 LC and 12 HC (*n* = 36). To account for high levels of expected attrition, 36 LC and 15 HC participants were recruited; however, only (total) 45 completed all visits. A 2:1 recruitment ratio was adopted to prioritize the investigation of the clinical population. This approach, which is common in clinical research (Peckham et al., 2015) allows for greater exploration of within‐group variability, which is a critical consideration in populations with diverse symptom profiles such as long COVID.

### 2.11 Statistical analysis

Due to logistical constraints, namely that a single researcher conducted both data collection and the primary statistical analysis, blinding of the researcher during analysis was not feasible. Descriptive statistics of continuous measures are presented as means and standard deviations unless otherwise stated. Frequency counts and percentages are reported for categorical variables associated with participant demographics. To compare demographic and habitual physical activity outcomes between LC and controls at baseline, independent samples *t*‐tests were used. Cohen's *d* effect sizes were reported and referred to as small (*d* = 0.2), moderate (*d* = 0.5), and large (*d* = 0.8). Analysis of covariance (ANCOVA) was used to compare baseline vascular measures between LC and controls. BMI was included as a covariate in the ANCOVA models where it was a significant predictor (*p* < 0.05) due to its influence on haemodynamic variables (Landi et al., 2018). Partial eta squared were reported and effect sizes are referred to as small ($\eta_{p}^{2}$ = 0.01), moderate ($\eta_{p}^{2}$ = 0.06), and large ($\eta_{p}^{2}$ = 0.14). To assess the effect of Condition (continuous sitting vs. interrupted sitting), between Groups (LC vs. healthy control), across Time (baseline vs. Post), linear mixed‐effects models were used for all vascular outcome measures. Originally, a three‐way model was used (Condition × Group × Time). Analyses then included two‐way models (Time × Condition; Time × Group) to assess variables. Fixed effects included group, condition, and time, which were assessed for main effects and their interactions. Participant ID was included as a random intercept to account for the repeated measures nature of the design. This model allowed all available data to be retained without the need for case‐wise deletion, thereby improving efficiency and reducing bias. For cfPWV, AP, AIx, and Aix75, MAP was included as a covariate (Stoner et al., 2013, 2014; Townsend et al., 2015). All linear mixed effects data are presented as estimated marginal means and standard error.

## 3 RESULTS

### 3.1 Participant characteristics

Recruitment occurred between 21/06/2022 and 30/07/2024. An overview of participant recruitment and retention is presented in the flow diagram (Figure S1). Thirty individuals with medically diagnosed LC and 15 healthy controls were recruited for the study. There were no adverse events for either group. Participants demographics and habitual PA can be found in Table 1. BMI was significantly higher in the LC group (MD = 3.67 kg/m^2^ *p* = 0.032, *d = 0.705*). LC participants had significantly lower levels of habitual PA, demonstrated by reduced steps (MD = 5493 steps, *p* = <0.001, *d =* 1.857) and activity level (MD = 2.44 MET·h^−1^/day, *p* = <0.001, *d =* 2.003), and higher sedentary (MD = 131 mins, *p* = <0.001, *d =* 1.298) and sitting time (MD = 100 mins, *p* = 0.008, *d =* 1.069). Those with LC demonstrated significantly worse perceived health (EQ‐5D‐5L) compared to the control (MD = 21.25, *p* < 0.001, *d =* 1.255). The most commonly reported symptoms of those with LC were fatigue (76.6%), trouble concentrating (70%), thinking (70%) and sleeping (60%), and shortness of breath (60%) (Figure S2).

**TABLE 1:** Means (standard deviation) of participant demographic, habitual physical activity, and quality of life data.

	LC	HC	MD	*p*	Effect size (Cohen's *d*)
*N* (female)	30 (23)	15 (7)			
Age (y)	52.7 (12.3)	52.9 (11.2)	0.17	0.965	0.014
Height (cm)	168.6 (8.0)	171.7 (6.4)	3.17	0.191	0.423
Weight (kg)	83.3 (20.1)	75.2 (8.8)	8.11	0.144	0.473
BMI (kg/m^2^)	29.2 (6.1)	25.5 (3.0)	3.67	**0.032**	**0.705**
Steps (per day)^a^	5765.6 (2348.4)	11258.6 (3667.4)	5493.00	**<0.001**	**1.857**
Sedentary time (mins)^a^	641.2 (85.5)	535.0 (85.4)	131.22	**<0.001**	**1.298**
Sitting time (mins)^a^	547.0 (99.1)	446.9 (86.3)	100.10	**0.008**	**1.069**
Activity level (MET·h^−1^/day)^a^	32.5 (1.0)	35.1 (1.4)	2.44	**<0.001**	**2.003**
EQ‐5D‐5L (scored out of 100)	59.5 (19.7)	80.8 (8.1)	21.25	**<0.001**	**1.255**
Time since infection (months)	27.1 (12.7)	N/A			

*Note*: Bold: Significant difference between groups. *n* = 45.

Abbreviations: BMI, body mass index; HC, healthy control; LC, long COVID; MET·h^−1^/day; metabolic equivalents per hour per day.

^a^ *n* = 29.

Baseline vascular health outcomes between groups are presented in Table S3. pDBP (77.7 mmHg SE = 1.0 vs. 72.8 mmHg SE = 1.6 *p* = 0.019 $\eta_{p}^{2}$ = 0.063), cDBP (78.2 mmHg SE = 1.1 vs. 73.6 mmHg SE = 1.5 *p* = 0.012 $\eta_{p}^{2}$ = 0.072), HR (67.6 bpm, SE = 1.1 vs. 55.9 bpm, SE = 1.6 p < 0.001, $\eta_{p}^{2}$ = 0.301), MAP (91.5 mmHg SE = 1.3 vs. 85.8 mmHg SE = 1.8, *p* = 0.013, $\eta_{p}^{2}$ = 0.070) AIx (23.7% SE = 1.5 vs. 16.3% SE = 2.2, *p* = 0.007, $\eta_{p}^{2}$ = 0.080), and AIx75 (19.2% SE = 1.5 vs. 9.1% SE = 2.2, *p* < 0.001, $\eta_{p}^{2}$ = 0.136) were significantly higher in the LC group compared to healthy controls.

### 3.2 Effect of prolonged sitting on vascular health

There were no three‐way (Time × Condition × Group) or two‐way (Time × Condition; Time × Group) interactions for any variable (Table 2; Table S3).

**TABLE 2:** Three‐way and two‐way interactions in response to uninterrupted and interrupted sitting.

Outcome	Group	Condition	Time		*p* Value		
			Pre	Post	3‐level	Time × group	Time × condition
			Mean (SE)	Mean (SE)			
cfPWV^a^, ^b^	LC	Un	7.4 (0.2)	7.5 (0.2)	0.535	0.118	0.521
		In	7.3 (0.2)	7.6 (0.2)			
	HC	Un	7.7 (0.3)	7.7 (0.3)			
		In	7.6 (0.3)	7.6 (0.3)			
pSBP	LC	Un	124.6 (2.7)	129.8 (2.7)	0.713	0.987	0.544
		In	125.5 (2.7)	128.6 (2.7)			
	HC	Un	117.6 (3.8)	122.1 (3.8)			
		In	118.1 (3.8)	122.1 (3.8)			
pDBP	LC	Un	77.6 (1.6)	81.8 (1.6)	0.579	0.365	0.359
		In	78.7 (1.6)	81.3 (1.6)			
	HC	Un	72.0 (2.3)	74.7 (2.3)			
		In	71.4 (2.3)	73.6 (2.3)			
pPP	LC	Un	47.0 (1.6)	47.7 (1.6)	0.986	0.375	0.957
		In	45.8 (1.6)	46.6 (1.6)			
	HC	Un	44.7 (2.3)	47.0 (2.3)			
		In	46.3 (2.3)	48.7 (2.3)			
cSBP	LC	Un	113.7 (2.5)	117.9 (2.5)	0.705	0.725	0.400
		In	115.0 (2.5)	116.9 (2.5)			
	HC	Un	106.5 (3.5)	110.6 (3.5)			
		In	106.9 (3.5)	110.2 (3.5)			
cDBP	LC	Un	78.1 (1.6)	82.5 (1.6)	0.926	0.177	0.356
		In	79.1 (1.6)	82.3 (1.6)			
	HC	Un	72.6 (2.3)	75.3 (2.3)			
		In	72.3 (2.3)	74.0 (2.3)			
cPP	LC	Un	35.1 (1.4)	35.2 (1.4)	0.897	0.155	0.584
		In	35.3 (1.4)	34.8 (1.4)			
	HC	Un	33.1 (2.1)	35.5 (2.1)			
		In	34.5 (2.1)	35.9 (2.1)			

*Note*: 3‐Level: Indicates values for the three‐way interaction of Time × Group × Condition. *N* = 45.

Abbreviations: cDBP, central diastolic blood pressure; cfPWV, carotid‐femoral pulse wave velocity; cPP, central pulse pressure; cSBP, central systolic blood pressure; HC, healthy control; LC, long COVID; pDBP, peripheral diastolic blood pressure; pPP, peripheral pulse pressure; pSBP, peripheral systolic blood pressure; RM, reflection magnitude.

^a^ MAP included as covariate.

^b^ Data based on *n* = 40 due to outcome measure validity.

There were main effects of time (Table S1), with increases in pSBP (MD = 4.17 mmHg, SE = 1.04 mmHg, *p* = <0.001), pDBP (MD = 2.93 mmHg, SE = 0.55 mmHg, *p* = <0.001), cSBP (MD = 3.37 mmHg, SE = 0.93 mmHg, *p* = <0.001), cDBP (MD = 3.00 mmHg, SE = 0.58 mmHg, *p* = <0.001), MAP (MD = 2.87 mmHg, SE = 0.74 mmHg, *p* = <0.001), and Pf (MD = 0.92 mmHg, SE = 0.42 mmHg, *p* = 0.031). There was a significant decrease in AP (MD = −1.13 mmHg SE = 0.40, *p* = 0.006), AIx (MD = −3.10%, SE = 0.89%, *p* = <0.001), AIx75 (MD = −3.51%, SE = 0.92%, *p* = <0.001), and RM (MD = −2.08%, SE = 0.74%, *p* = 0.006). There was no time effect for cfPWV (MD = −0.13, SE = 0.09, *p* = 0.170).

## 4 DISCUSSION

The present study demonstrates that individuals with long COVID (LC) exhibit similar vascular responses than that of healthy controls (HC) following acute periods of uninterrupted sitting, and light PA is not sufficient to mitigate BP responses to sitting.

Previous research has highlighted persistent endothelial dysfunction and arterial stiffness as consequences of SARS‐CoV‐2 infection (Jannasz et al., 2023; Oikonomou et al., 2022). Studies have demonstrated increased PWV and reduced flow‐mediated dilation (FMD) in individuals with LC, suggesting prolonged vascular impairments (Mclaughlin et al., 2025; Nandadeva et al., 2023). However, the present findings indicate that, at least in response to acute sitting, vascular function in individuals with LC does not appear to be significantly different from that of healthy controls. One possible explanation for this discrepancy is the timeline of vascular recovery post‐infection. While early evidence suggested endothelial dysfunction and impaired autonomic regulation in LC patients (Marques et al., 2023; Oikonomou et al., 2022), our findings imply that these impairments may not persist beyond 2 years post‐infection or may not translate to acute challenges such as prolonged sitting. It is also possible that adaptations occur over time, allowing vascular responses to normalize, particularly in individuals without severe ongoing symptoms. Moreover, prolonged SB is known to impair vascular health, particularly by reducing shear stress and nitric oxide availability (Paterson et al., 2020; Pekas et al., 2023). Given that LC is associated with increased SB (Wright et al., 2022), it was plausible that this population would exhibit heightened vulnerability to acute sitting‐induced vascular dysfunction. However, our results do not support this hypothesis, suggesting that other compensatory mechanisms may counteract these effects.

### 4.1 Effect of uninterrupted sitting

Sitting periods had no effect on arterial stiffness (AS), which conflicts with current research demonstrating that uninterrupted sitting causes an acute increase in AS. One possible explanation for these contrasting findings is the difference in sitting time. Most acute sitting research typically employs 3‐h sitting periods, where responses are likely more pronounced due to factors such as increased venous pooling and the lower metabolic cost of sitting compared to standing or light physical activity, especially over extended durations (Júdice et al., 2016; Paterson et al., 2024). In contrast, 2 h is the upper limit of common habitual sitting durations (O'Brien et al., 2022), and was deemed representative and feasible by study PPIE contributors. Our accelerometer data suggests that individuals with long COVID spent more time in SB than HC's. As such, it may be that a 2‐h sitting period may not represent a meaningful departure from their habitual behavior, potentially attenuating any physiological response. This interpretation aligns with recent work by Paterson et al. (2024), suggesting that 3 h of sitting may represent a minimal threshold for detecting acute changes in cfPWV.

Acute, prolonged sitting resulted in significant increases in central (Mean difference (SE); cSBP = 3.37 mmHg (1.2); cDBP = 3.00 mmHg (0.8)) and peripheral blood pressure (pSBP = 4.17 mmHg (1.04); pDBP = 2.93 mmHg (0.55)), and MAP (2.87mHg (0.74)). These vascular responses were greater than those that have been previously reported (Adams et al., 2023; Paterson et al., 2024). A meta‐analysis described modest increases of 0.42 mmHg/h for peripheral systolic and 0.24 mmHg/h for peripheral diastolic pressures during uninterrupted sitting (Adams et al., 2023). Our results indicate over a fivefold greater increase in BP, consistent across both LC and control groups compared to Adams' review. However, the review does not specify the posture in which hemodynamic measurements were taken, despite posture being a known predictor of BP (Jamieson et al., 1990). Since the studies included in the review report both supine and seated measurements, it is difficult to draw clear conclusions. Additionally, Paterson's study, for example, which clearly describes supine measures, reported a smaller increase in pSBP after 2 h sitting (2.50 ± 1.33 mmHg) compared to our seated BP measurements (Paterson et al., 2024). The difference in posture could have masked a larger change in pressure, as our findings indicated no time effect of BP in the supine position. Research has sought to standardize procedures in sitting studies (Paterson et al., 2023), and measuring BP in a seated position avoids additional orthostatic challenges when moving to a supine position disrupting the results.

Acute increases in BP are thought to be driven by blood pooling in the lower limbs during sitting. Although this study did not directly measure pooling, several studies have documented increases in calf circumference (Fryer et al., 2021) or changes in gastrocnemius oxygenation (Fryer et al., 2021) as indicators of pooling following sitting periods. This pooling reduces venous return, leading to decreased cardiac output and shear stress, which can contribute to acute endothelial dysfunction and potential aortic stiffening (although not evident in this study), driving an increase in BP. Reduced cardiac output may also lower renal perfusion, stimulating the renin‐angiotensin‐aldosterone system (RAAS), which further increases BP. Although this increase in BP was observed similarly in both groups, inflammation‐related endothelial damage, particularly in LC patients (Bielecka et al., 2024), might exacerbate these effects during longer sitting periods by disrupting the RAAS.

### 4.2 Effect of interrupted sitting

The present study demonstrated no significant three‐way (Condition by Group by Time) or two‐way interaction for Condition by Time for any variable, suggesting that interrupting sitting with light bouts of movement has no effect on sitting‐induced hemodynamic changes. While previous research has reported benefits of breaking up prolonged sitting, differences in study design may explain this disparity (Paterson et al., 2022). The frequency, duration, and intensity of movement interruptions are known to influence vascular responses, and it is possible that the specific protocol used (simple resistance activities (calf raises, sit‐to‐stands) combined with aerobic movement (walking), totalling ~4 min per break) was not sufficient to counteract the haemodynamic effects of prolonged sitting in this sample, despite being informed by prior literature and PPIE. Specifically, a greater sitting period or greater LPA was highlighted by PPIE as potentially detrimental for participants. The current sitting time and LPA accurately reflected the literature and patient safety concerns.

A meta‐analysis by Paterson et al., (2022) demonstrates that interrupting prolonged sitting, particularly with aerobic activity, has a protective effect against sitting‐induced increases in blood pressure, with the greatest benefits observed in protocols featuring frequent, longer (≥5 min), or higher‐intensity interruptions. This suggests that while breaking up sitting remains a promising strategy, the magnitude and type of movement required to elicit meaningful vascular benefits may be greater than what was implemented in the present study. Future research should explore whether more prolonged or intense movement breaks, or alternative strategies such as standing desks, could offer more effective countermeasures against the hemodynamic consequences of prolonged sitting.

### 4.3 Implications

The findings suggest that individuals with long COVID are not disproportionately vulnerable to the acute vascular effects of prolonged sitting compared to controls. This indicates that, in the context of short‐term sitting periods, targeted SB interventions may not be necessary for this population. However, the absence of a protective effect from LPA interruptions highlights the potential limitations of low‐intensity movement in mitigating sitting‐induced increases in blood pressure. Future research should explore longer uninterrupted sitting periods that better reflect habitual SB in individuals with long COVID, as well as investigate vascular responses in those closer to the acute phase of infection, where pathophysiological changes may be more pronounced.

### 4.4 Strengths and limitations

The LC group had a higher proportion of female participants (77%) compared to the healthy controls (HC) group (47%). Although the literature suggests a higher incidence of LC in females (Subramanian et al., 2022; Walker et al., 2021), our overall sample had a large percentage of female participants (67%). We did not collect data on menstrual cycle or menopausal status; however, existing research indicates that menopausal status does not significantly influence vascular responses to prolonged sitting (Moinuddin et al., 2024).

Our data reports baseline vascular health, using BMI, a significant predictor of many vascular variables as a covariate. Currently, due to the novel nature of the condition, it is uncertain whether changes to the vascular system are a result of viral infection or deconditioning/long‐term increases in SB due to LC. Therefore, it should be noted that raw data demonstrated significantly higher additional blood pressures in those with LC, supportive of current literature (Nandadeva et al., 2023).

Although care was taken to obtain complete datasets, some missing data occurred, primarily related to the use of activity monitors. Specifically, a subset of participants (*n* = 16) did not yield valid activPAL recordings, leading to a reduced sample size for analyses of habitual activity. Missing data were predominantly due to device malfunction. In addition, five participants were excluded from cfPWV analysis due to an inability to obtain valid measures using the SphygmoCor XCEL device. These occurrences are recognized limitations when working with operator‐dependent assessments in clinical populations and should be considered when interpreting the findings.

## 5 CONCLUSION

The present study demonstrates that individuals with long COVID (LC) exhibit similar vascular responses to prolonged uninterrupted sitting, which increases similarly; however, AS is unchanged. Interrupting sitting with LPA did not mitigate sitting‐induced increases in BP regardless of LC diagnosis. The findings suggest that individuals with long COVID are not disproportionately vulnerable to the acute vascular effects of prolonged sitting compared to controls.

## FUNDING INFORMATION

This research was supported by the University of Winchester Research and Innovation fund.

## CONFLICT OF INTEREST STATEMENT

The authors declare that they have no conflict of interest.

## ETHICS STATEMENT

The study received approval from the HRA and Health and Care Research Wales (HCRW), reference 22/SC/0120.

## Supporting information


Appendix S1.

Supplementary Data.

## Data Availability

The data that support the findings of this study are available as supporting information in the online version of the article. The data can be accessed in the supplementary files.
