# Correction to “The effect of uninterrupted and interrupted sitting on vascular function in adults with long COVID”

**DOI:** 10.14814/phy2.70658

**Published:** 2025-11-20

Hudson, N., Hannah, S., Husted, M., Fryer, S., Ryan‐Stewart, H., Rickenbach, M., Stone, K., & Faulkner, J. (2025). The effect of uninterrupted and interrupted sitting on vascular function in adults with long COVID. *Physiological Reports*, *13*(19), e70452.

The authors regret that certain errors in numerical values were not corrected at proof stage. In section 3.2 paragraph 2, the text currently reads: “There was no time effect for cfPWV (MD = −0.13, SE = 0.09, *p* = 0.170).”

It should read: “There was no time effect for cfPWV (MD = 0.13, SE = 0.09, *p* = 0.170).”

In section 3.1 paragraph 2, Table S3 is mistakenly referenced. The reference should be to Table S2.

In section 3.1 paragraph 2, the text reads “pDBP (77.7 mmHg, SE = 1.0 vs. 72.8 mmHg, SE = 1.6, *p* = 0.019, $\eta_{p}^{2}$ = 0.063), cDBP (78.2 mmHg, SE = 1.1 vs. 73.6 mmHg, SE = 1.5, *p* = 0.012, $\eta_{p}^{2}$ = 0.072).”

It should read: “pDBP (77.7 mmHg, SE = 1.0 vs. 72.8 mmHg, SE = 1.5, *p* = 0.012, $\eta_{p}^{2}$ = 0.072), cDBP (78.2 mmHg, SE = 1.1 vs. 73.6 mmHg, SE = 1.6, *p* = 0.019, $\eta_{p}^{2}$ = 0.063).”

In Table S2, the *p* value for pDBP reads as “<0.012.” This should read “0.012.”

We apologize for these errors. The changes do not affect the scientific conclusions of the study or any of the interpretations in the paper.

