# Supplementary material for: The effect of uninterrupted and interrupted sitting on vascular function in adults with long COVID
Source: Physiol Rep. 2025 Oct 6;13(19):e70452. doi: 10.14814/phy2.70452 (PMC13045372; doi:10.14814/phy2.70452)
Supplement: Supplementary file 1 — Appendix S1. [file PHY2-13-e70452-s002.docx]

**Supplementary Tables and Figures**

*
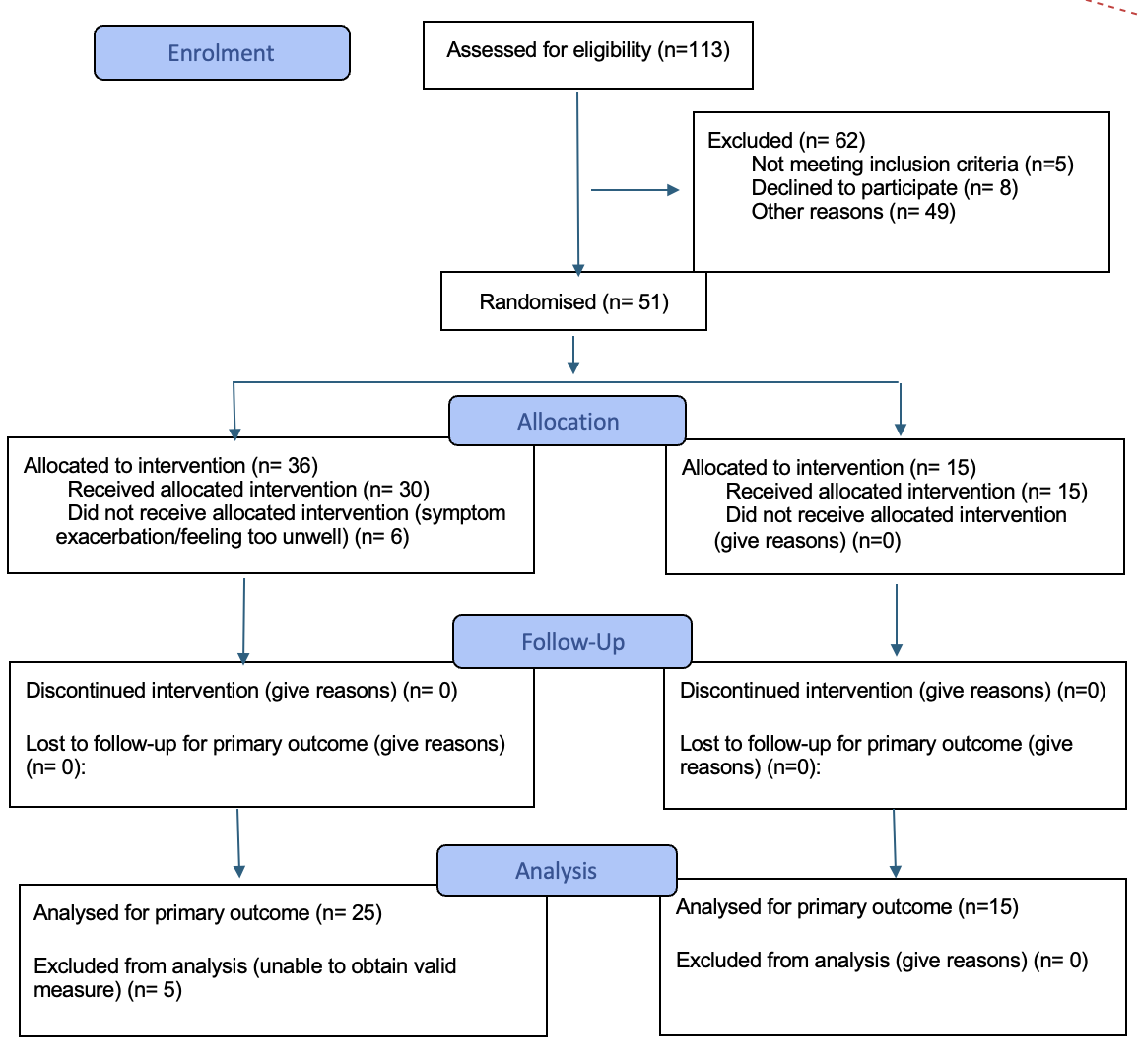
*

***Figure S1:*** *CONSORT 2025 Flow Diagram*

Flow diagram of the progress through the phases of a randomised trial of two groups (that is, enrolment, intervention allocation, follow-up, and data analysis)

**Table S1** Seated main effect of time. Data are reported as means and standard error in response to prolonged sitting

| Measure | 10 min | 120 min | MD (SE) | Main effect Time  *p* |
| --- | --- | --- | --- | --- |
| pSBP (mmHg) | 121.5 (2.2) | 125.6 (2.2) | 4.17 (1.04) | **<0.001** |
| pDBP (mmHg) | 74.9 (1.3) | 77.9 (1.3) | 2.93 (0.55) | **<0.001** |
| pPP (mmHg) | 45.9 (1.3) | 47.5 (1.3) | 1.58 (0.92) | 0.087 |
| cSBP (mmHg) | 110.5 (2.0) | 113.9 (2.0) | 3.37 (0.93) | **<0.001** |
| cDBP (mmHg) | 75.3 (1.4) | 78.5 (1.4) | 3.00 (0.58) | **<0.001** |
| cPP (mmHg) | 35.1 (1.3) | 34.7 (1.8) | 0.85 (0.74) | 0.253 |
| AP (mmHg)* | 8.0 (0.8) | 6.9 (0.8) | -1.13 (0.40) | **0.006** |
| AIx (%)* | 20.8 (1.8) | 17.7 (1.7) | -3.10 (0.89) | **<0.001** |
| AIx75 (%)* | 14.8 (1.7) | 11.3 (1.6) | -3.51 (0.92) | **<0.001** |
| HR (bpm) | 61.8 (1.4) | 61.5 (1.4) | -0.25 (0.76) | 0.738 |
| MAP (mmHg) | 88.1 (1.6) | 91.00 (1.6) | 2.87 (0.74) | **<0.001** |
| Pf (mmHg) | 24.3 (0.7) | 25.2 (0.7) | 0.92 (0.42) | **0.031** |
| Pb (mmHg) | 14.5 (0.6) | 14.5 (0.6) | 0.02 (0.34) | 0.961 |
| RM (%) | 59.9 (1.5) | 57.8 (1.5) | -2.08 (0.74) | **0.006** |

*Measures of Pulse Wave Analysis at 10 min and 120 min. Measures recorded in the seated position. Analysis is irrespective of group or condition. AIx; augmentation index, AIx75; augmentation index normalised to HR 75bpm; AP; augmentation pressure, cDBP; central diastolic blood pressure, cPP; central pulse pressure, HR; Heart rate, Pb; reflected wave component, pDBP; peripheral diastolic blood pressure, Pf; forward wave component, pPP; peripheral pulse pressure, pSBP; peripheral systolic blood pressure, RM; reflection magnitude. Bonferroni post hoc analysis used to identify statistical significant between groups. n=45*

*Bonferroni post hoc analysis used to identify statistical significant between groups. Bold indicates p ≤ 0.05*

** MAP included as covariate*

**Table S2** ANCOVA means for Group Differences in vascular measures at baseline

|  | LC (Means/SE) | HC (Means/SE) | *p* | *Effect Size*  *η_p_^2^* |
| --- | --- | --- | --- | --- |
| cfPWV (m/s­^-1^)^#^ | 7.5 (0.2) | 7.4 (0.3) | 0.761 | 0.001 |
| pSBP (mmHg)* | 125.7 (1.7) | 123.0 (2.4) | 0.341 | 0.011 |
| pDBP (mmHg)* | 77.7 (1.0) | 72.8 (1.5) | **<0.012** | **0.072** |
| pPP (mmHg)* | 45.5 (1.1) | 47.4 (1.5) | 0.287 | 0.013 |
| cSBP (mmHg)* | 113.2 (1.6) | 109.3 (2.2) | 0.164 | 0.023 |
| cDBP (mmHg)* | 78.2 (1.1) | 73.6 (1.6) | **0.019** | **0.063** |
| cPP (mmHg)* | 34.5 (0.9) | 35.3 (1.3) | 0.639 | 0.003 |
| AP (mmHg)* | 8.5 (0.7) | 6.6 (1.1) | 0.145 | 0.024 |
| AIx (%) | 23.7 (1.5) | 16.3 (2.2) | **0.007** | **0.080** |
| AIx75 (%)* | 19.2 (1.5) | 9.1 (2.2) | **<0.001** | **0.136** |
| MAP (mmHg)* | 91.5 (1.3) | 85.8 (1.8) | **0.013** | **0.070** |
| HR (mmHg) | 67.6 (1.1) | 55.9 (1.6) | **<0.001** | **0.301** |
| Pf (mmHg)* | 24.8 (0.5) | 24.4 (0.8) | 0.686 | 0.002 |
| Pb (mmHg)* | 14.7 (0.4) | 14.5 (0.6) | 0.779 | 0.001 |
| RM (%) | 60.4 (1.3) | 59.4 (1.9) | 0.657 | 0.002 |

*AIx; augmentation index, AIx75; augmentation index normalised to HR 75bpm; AP; augmentation pressure, cDBP; central diastolic blood pressure, cPP; central pulse pressure, Pb; reflected wave component, pDBP; peripheral diastolic blood pressure, Pf; forward wave component, pPP; peripheral pulse pressure, pSBP; peripheral systolic blood pressure, cfPWV; carotid-femoral pulse wave velocity, RM; reflection magnitude, LC; Long COVID, HC; Healthy Control. Bonferroni post hoc analysis used to identify statistical significance between groups. n=45*

*Bold: Significant difference between groups*

** BMI used as a covariate*

*^#^ data based on n = 40 due to outcome measure validity*


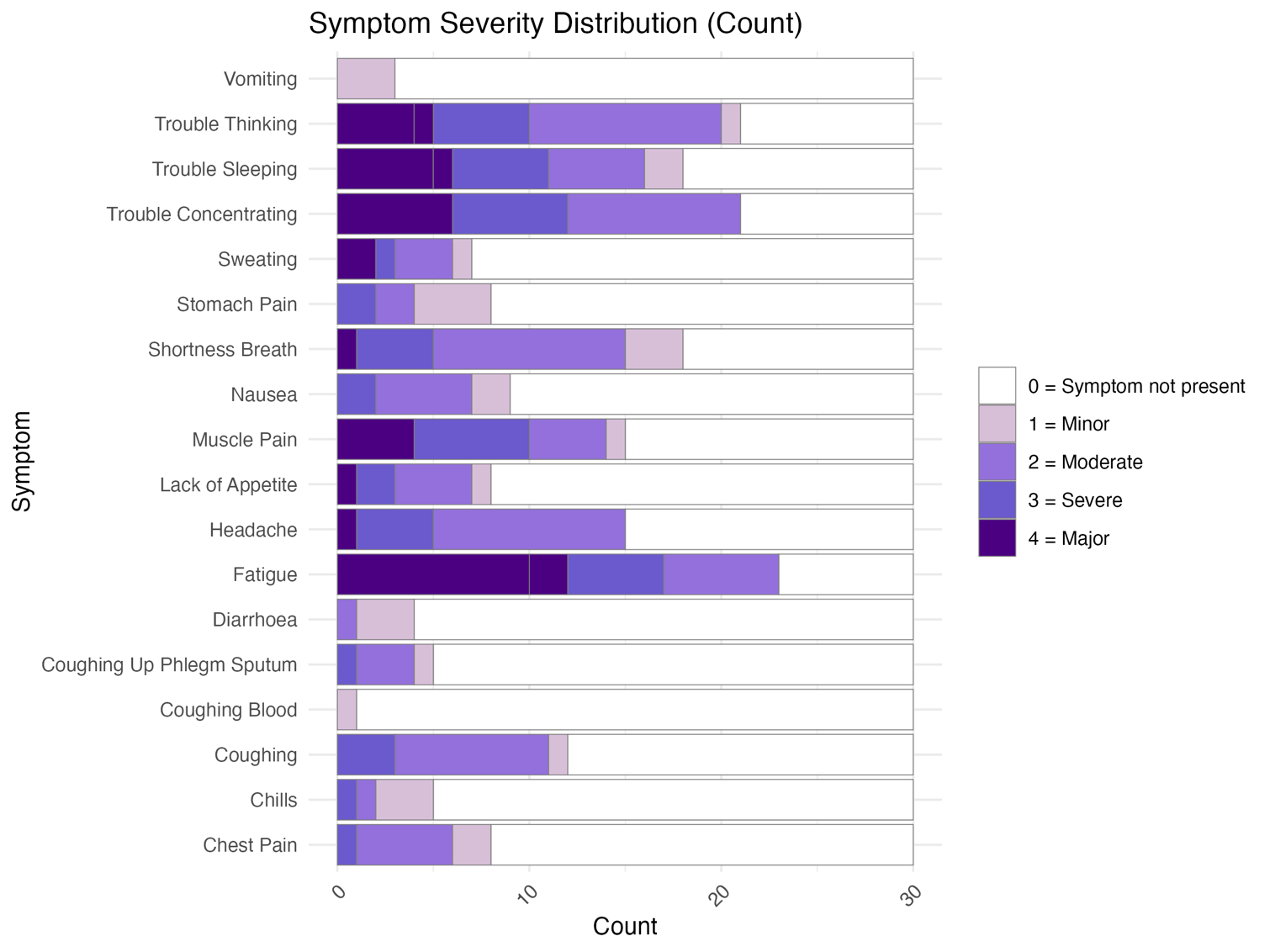
**Figure S2:** COVID-19 Symptom score; n=30

**Table S3** Three-way and two-way interactions in response to uninterrupted and interrupted sitting for vascular variables

| **Outcome** | **Group** | **Condition** |  | **Time** | | | |  | **P Value** | | | | |
| --- | --- | --- | --- | --- | --- | --- | --- | --- | --- | --- | --- | --- | --- |
|  |  |  |  | **Pre** | | **Post** | |  | **3-Level** | **Time x**  **Group** | | **Time x Condition** | |
|  |  |  |  | **Mean (SE)** | | **Mean (SE)** | |  |  | |  | |  |
| AP* | LC | Un |  | 8.1 | (1.0) | 6.8 | (1.0) |  | 0.330 | 0.071 | | 0.874 | |
|  |  | In |  | 9.1 | (1.0) | 6.8 | (1.0) |  |  |  |  |  |  |
|  | HC | Un |  | 7.3 | (1.4) | 6.6 | (1.4) |  |  |  |  |  |  |
|  |  | In |  | 7.6 | (1.4) | 7.5 | (1.4) |  |  | |  | |  |
| MAP | LC | Un |  | 91.7 | (2.0) | 96.1 | (2.0) |  | 0.633 | 0.416 | | 0.404 | |
|  |  | In |  | 92.8 | (2.0) | 95.3 | (2.0) |  |  |  |  |  |  |
|  | HC | Un |  | 84.0 | (2.8) | 86.5 | (2.8) |  |  |  |  |  |  |
|  |  | In |  | 84.0 | (2.8) | 86.0 | (2.8) |  |  | |  | |  |
| AIx* | LC | Un |  | 21.7 | (2.1) | 18.6 | (2.2) |  | 0.186 | 0.106 | | 0.752 | |
|  |  | In |  | 24.9 | (2.1) | 19.1 | (2.1) |  |  |  |  |  |  |
|  | HC | Un |  | 18.8 | (3.1) | 16.2 | (3.0) |  |  |  |  |  |  |
|  |  | In |  | 17.9 | (3.1) | 17.0 | (3.0) |  |  | |  | |  |
| Aix75* | LC | Un |  | 17.8 | (2.0) | 15.3 | (2.1) |  | 0.060 | 0.247 | | 0.679 | |
|  |  | In |  | 21.6 | (2.0) | 15.0 | (2.1) |  |  |  |  |  |  |
|  | HC | Un |  | 10.7 | (2.9) | 7.0 | (2.9) |  |  |  |  |  |  |
|  |  | In |  | 9.2 | (2.9) | 8.0 | (2.9) |  |  | |  | |  |
| HR | LC | Un |  | 66.9 | (1.7) | 68.0 | (1.7) |  | 0.65 | 0.747 | | 0.373 | |
|  |  | In |  | 68.3 | (1.7) | 67.3 | (1.7) |  |  |  |  |  |  |
|  | HC | Un |  | 55.6 | (2.4) | 55.4 | (2.4) |  |  |  |  |  |  |
|  |  | In |  | 56.3 | (2.4) | 55.5 | (2.4) |  |  | |  | |  |
| Pf | LC | Un |  | 24.8 | (0.9) | 25.6 | (0.9) |  | 0.449 | 0.276 | | 0.992 | |
|  |  | In |  | 25.8 | (0.9) | 25.9 | (0.9) |  |  |  |  |  |  |
|  | HC | Un |  | 23.2 | (1.2) | 24.3 | (1.2) |  |  |  |  |  |  |
|  |  | In |  | 23.5 | (1.2) | 25.2 | (1.2) |  |  |  |  |  |  |
| Pb | LC | Un |  | 14.9 | (0.7) | 14.9 | (0.7) |  | 0.731 | 0.694 | | 0.922 | |
|  |  | In |  | 15.0 | (0.7) | 14.8 | (0.7) |  |  |  |  |  |  |
|  | HC | Un |  | 13.9 | (1.0) | 13.9 | (1.0) |  |  |  |  |  |  |
|  |  | In |  | 14.0 | (1.0) | 14.3 | (1.0) |  |  |  |  |  |  |
| RM | LC | Un |  | 61.1 | (1.9) | 58.1 | (1.9) |  | 0.353 | 0.440 | | 0.712 | |
|  |  | In |  | 59.7 | (1.9) | 57.5 | (1.9) |  |  |  | |  | |
|  | HC | Un |  | 59.9 | (2.7) | 59.4 | (2.7) |  |  |  | |  | |
|  |  | In |  | 58.9 | (2.7) | 56.4 | (2.7) |  |  |  | |  | |

*AIx; augmentation index, AIx75; augmentation index normalised to HR 75bpm; AP; augmentation pressure, Pb; reflected wave component, , LC; Long COVID, HC; Healthy Control, Pf; forward wave component, cfPWV; carotid-femoral pulse wave velocity, RM; reflection magnitude. 3-Level: Indicates values for the three-way interaction of Time x Group x Condition. N = 45*

** MAP included as covariate*
